# The data on the aerobic training with or without calorie restriction and muscular levels of Irisin and muscular FNDC5 concentration in obese male Wistar rats

**DOI:** 10.1016/j.dib.2018.10.028

**Published:** 2018-10-22

**Authors:** Hossein Shirvani, Alireza Delpasand, Behzad Bazgir

**Affiliations:** Exercise Physiology Research Center, Life Style Institute, Baqiyatallah University of Medical Sciences, Tehran, Iran

**Keywords:** Training, Animal model, Diet, Exercise

## Abstract

The present data article aims at investigating the muscular levels of Irisin, FNDC5, and UCP1 in male Wistar rats during the aerobic exercise with or without calorie restriction (CR). Twenty four, 8-week-old male Wistar rats (190±16 g) were selected and purchased for the research. After obesity induction by high-fat diet, the animals were randomly divided into three groups: exercise EX (*n* = 8), EX-CR (*n* = 8) and CO as control (*n* = 8). EX exercised 6 sessions per week and EXCR exercise 3 sessions + 3 days caloric restriction per week. The Irisin (Cat.No:CK-E91266 & Intra-Assay: CV<10%), FNDC5 (Cat.No:CK-E91393 & Intra-Assay: CV<10%) levels were assessed by the special Rat ELISA Kit (EASTBIOPHARM, China, under licensed by the United States). Muscular Irisin concentrations in EX group were higher than other groups. In addition, FNDC5 concentrations in EX group was higher than those in other groups.

**Specifications table**

**Table:** 

Subject area	Physiology
More specific subject area	Exercise Physiology
Type of data	Figures
How data was acquired	This data was acquired from twenty four, 8 week-old male Wistar rats (190±16 g) categorized into three groups.
Data format	Raw and analyzed
Experimental factors	Soleus muscle were promptly gathered and frozen by immersion in liquid nitrogen, and stored at −80 °C until analyzed.
Experimental features	The Irisin (Cat.No:CK-E91266 & Intra-Assay: CV<10%), FNDC5 (Cat.No:CK-E91393 & Intra-Assay: CV<10%) and UCP1 (Cat.No:CK-E91312 & Intra-Assay: CV<10%) levels were assessed by the special Rat ELISA Kits.
Data source location	Tehran, Iran
Data accessibility	Data are included in this article
Related research article	D.M. Samy, C.A. Ismail, R.A. Nassra, Circulating irisin concentrations in rat models of thyroid dysfunction—effect of exercise, Metabolism.64(2015)804-13 [1].

**Value of the data**

• This data provides data on the exercise and obesity, where the evidences are still limited [2], [3].
• This data would be interesting for other researchers working on exercise in animal models.
• This data can be useful in the prevention and treatment of obesity.

## 1 Data

**Fig. 1:**
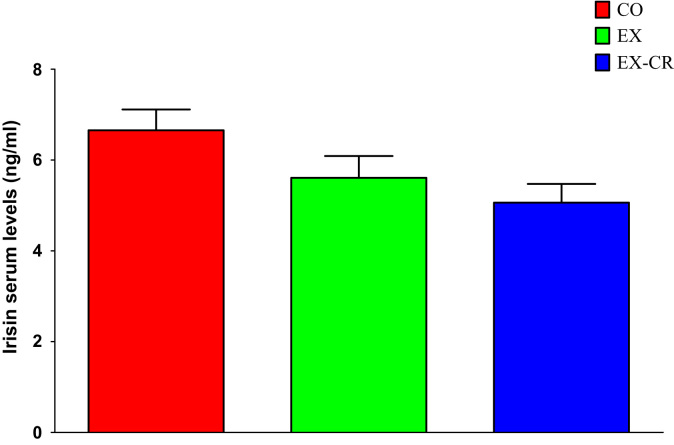
Irisin serum levels in three rats groups (EX: Exercise, EX-CR: exercise combined with 10 percent calorie restriction, CO: control group).

**Fig. 2:**
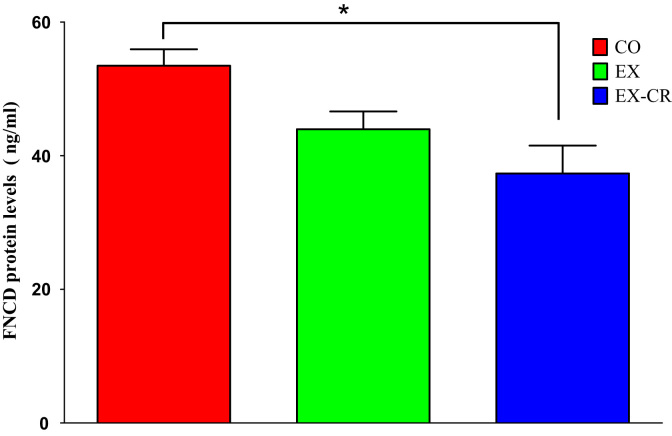
FNDC protein levels in skeletal muscle of three rat groups (EX: Exercise, EX-CR: exercise combined with 10 percent calorie restriction, CO: control group) * : significant differences in compare to control group.

Average (±standard deviation) of Irisin concentrations are shown in Fig. 1. In addition, the average (±standard deviation) of FNDC5 concentrations are illustrated in Fig. 2. Table 1 presents the values of UCP-1 in the rat groups.

**Table 1:** The UCP-1 values in three rat groups.

	**EX**	**EXCR**	**CO**
UCP-1 (ng/ml)	5.53 ± 0.66	5.28 ± 0.81	4.35 ± 0.96

EX: exercise group, EXCR: exercise combined with 10 percent calorie restriction group, CO: control group.

## 2 Experimental design, materials and methods

Twenty four, 8 week-old male Wistar rats (190±16 g) were chosen for the research. All the rats were housed in the following conditions: in temperature (23±2 °C) and cages (*n* = 5) under controlled dark/light cycle (12/12 h). The rats were acclimated to laboratory conditions for seven days before protocol initiation. After obesity induction, obesity group were randomly divided into three groups: EX (*n* = 8), EX+CR (*n* = 8) and CO as control (*n* = 8). Exercise training and caloric restriction protocol were approved by the post-graduate committee of Ferdowsi University of Mashhad. Other experimental procedures involving animals were approved by Ferdowsi University of Mashhad Animal Care and Use Committee on the Ethics: (permit code: 3.40388). All surgeries were executed under sodium pentobarbital anesthesia, and all endeavors were made to lower animal suffering.

The animals fed palate containing more energy and fat than standard regular food (4.8 vs. 3.9 kcal and 39% vs. 3.5% fat). All of the rats fed high fat palate for 17 wks. For the reason, the BMI for normal rats ranges between 0.45 and 0.68 g/cm^2^, the Rats BMI reached (0.70±17) and regarded as obese subjects (https://www.caymanchem.com/Article/2192). For making the protocol iso-caloric, rats daily calorie intake (DCI) were measured by weighting food intake (FI) of each group for seven days multiplying by palate calorie per gr provided by manufacturer Company (Behparvar. CO). Afterwards, DCI was multiplied by 10/100 (10%).

In EX group exercise session designed equal to 10% of their DCI. EXCR had both exercise and CR every other day in week. 3 day for exercise and 3 day for CR.

The EX and EXCR were exercised on a treadmill 6 and 3 days per week and every other day respectively. The exercise began between 7 to 11 a.m. in an approximately dark room. During the 1st adaptation week, the rats ran at speed of 10 m/min for 15 min up to 28 m/min. For encouragements of Rats, a mild electric shock was used. Then, the animals ran at speed of 28 m/min (70–75% vo2max) and duration of (55 min for EX), 46 min for EXCR for 8 weeks. The duration was equal to 10% of each group DCI.

Exercise sessions designed equal to 10% of DCI through shepherd and Gollnick equations:

Resting oxygen (2.42 O2 (100 g × min)^−1^) – exercise oxygen (7.77 ml (100 g × min)^−1^ in speed of 28 m/min) × 4.86 kcal/l O2 (assuming an RQ of 0.85) = calorie expenditure during treadmill running.

Primarily, subjects accessed to a high magnitude of primary food (P F) weighted. Then after a day, the residual palate (RF) was weighted and it was repeated for 7 days and the primary FI was measured by subtraction of PF from RF. Then mean FI was multiplied by 10 percent and was subtracted from mean FI shown below:

$$\begin{array}{ccc} FI\;\left(g\right) & \;= & \;PF\;\left(g\right)\;-\;RF\;\left(g\right)\\ Restricted\;FI & \;= & \;\left(10/100\times FI\;\left(g\right)\right)\;–\;FI\;\left(g\right) \end{array}$$

Nevertheless, palate energy was provided by manufacturer Company: 3.9 kcal per g. FI available to subjects was 14 g for EXCR. Food given to EX was 19 g without subtraction as they exercised equivalent to 10 percent of their DCI. In addition, a high amount of high-fat palate was available to obesity control until the protocol was over. The characteristics of palates are presented in Table 2.

**Table 2:** Palate content provided for rats.

**Contents**	**Standard**	**High-fat**
Protein	20	18
Fat	**3.5**	**39**
Carbohydrate	25	20
Fiber	15	2
Ash	10	10
Calcium	1	1
Phosphorus	0.7	0.7
Salt	0.5	0.5
Humidity	10	5
Lysine	1.15	1.15
Methionine	0.33	0.33
Met+sys	0.63	0.63
Thr+sys	0.95	0.95

The Rats were anesthetized with diethyl ether and sodium pentobarbital 50 mg/kg, intraperitoneal injection after a 12-h fast and 32 h following the last session of running, in order to highly decrease the effect of acute exercise session. Soleus muscle were promptly gathered and frozen by immersion in liquid nitrogen, and stored at −80 °C until analyzed.

To assess the Irisin muscular level, tissue collected from the soleus was poured into non-anticonvulsant tubes and immediately. Samples were centrifuged at 3000 rpm for 15 min at 4 °C temperature. After extraction of the sample from the freezer, they were mired and smashed, and then the powder was transferred to the homogenizer tube and mixed with the RIPA buffer solution (NaCl: 0.88 g, EDTA: 0.15 g, NP-40 or Triton X-100: 1 g, Sodium deoxycholate: 1 g, SDS: 0.10 g, diH2O: 80 ml, 1 M Tris–HCl, pH 7.6: 2.5 ml, diH2O to 100 ml, PMSF: 1 ml) and combined with a homogenizer (Potter Elvejheim) for 15 s later. All steps were taken in an ice cube circumstance. Finally, The microtube was centrifuged at the speed of 20,000 RPM at 4 °C for 20 min. The Irisin (Cat.No:CK-E91266 & Intra-Assay: CV<10%), FNDC5 (Cat.No:CK-E91393 & Intra-Assay: CV<10%) and UCP1 (Cat.No:CK-E91312 & Intra-Assay: CV<10%) levels were assessed by the special Rat ELISA Kit (EASTBIOPHARM, China, under licensed by the United States) and (UCP1 ELISA Kit No. ABIN435429).
