# Supplementary material for: The data on the aerobic training with or without calorie restriction and muscular levels of Irisin and muscular FNDC5 concentration in obese male Wistar rats
Source: Data Brief. 2018 Oct 22;21:888–92. doi: 10.1016/j.dib.2018.10.028 (PMC6223192; doi:10.1016/j.dib.2018.10.028)
Supplement: Supplementary file 1 — Transparency document. [file mmc1.pdf]

Author Declaration of Conflict of Interests

We wish to draw the attention of the Editor to the following facts which may be considered as potential conflicts of interest and to significant financial contributions to this work. [OR] We wish to confirm that there are no known conflicts of interest associated with this publication and there has been no significant financial support for this work that could have influenced its outcome.

We confirm that the manuscript has been read and approved by all named authors and that there are no other persons who satisfied the criteria for authorship but are not listed. We further confirm that the order of authors listed in the manuscript has been approved by all of us.

We confirm that we have given due consideration to the protection of intellectual property associated with this work and that there are no impediments to publication, including the timing of publication, with respect to intellectual property. In so doing we confirm that we have followed the regulations of our institutions concerning intellectual property.

We further confirm that any aspect of the work covered in this manuscript that has involved either experimental animals or human patients has been conducted with the ethical approval of all relevant bodies and that such approvals are acknowledged within the manuscript. [CAN BE DELETED IF NOT RELEVANT]

We understand that the Corresponding Author is the sole contact for the Editorial process (including Editorial Manager and direct communications with the office). He/she is responsible for communicating with the other authors about progress, submissions of revisions and final approval of proofs. We confirm that we have provided a current, correct email address which is accessible by the Corresponding Author and which has been configured to accept email from. (PLEASE CONFIRM WHAT E-MAIL ADDRESS YOU WISH TO INSERT HERE)

The Data on the Aerobic Training with or Without Calorie Restriction and Muscular Levels of Irisin and Muscular FNDC5 Concentration in Obese Male Wistar Rats

Hossein Shirvani [a], Alireza Delpasand [a], Behzad Bazgir [a,*]

[a] *Exercise Physiology Research Center, Life Style Institute, Baqiyatallah University of Medical Sciences, Tehran, Iran*

*** Corresponding author:** Behzad Bazgir
E-mail address: bazgirbehzad@bmsu.ac.ir (B. Bazgir)
Tel: (+98) 9107784079
